# Methodological Insights From Evaluating a Family Member's Voice Reorientation Programme: An Interpretive Descriptive Approach

**DOI:** 10.1002/nop2.70291

**Published:** 2025-08-31

**Authors:** Gideon U. Johnson, Amanda Towell‐Barnard, Christopher McLean, Glenn Robert, Beverley Ewens

**Affiliations:** ^1^ School of Nursing and Midwifery Edith Cowan University Joondalup Western Australia Australia; ^2^ Chelsea and Westminster Hospital NHS Foundation Trust London UK; ^3^ Florence Nightingale Faculty of Nursing, Midwifery and Palliative Care King's College London London UK; ^4^ Centre for Nursing Research, Sir Charles Gairdner Hospital Nedlands Western Australia Australia; ^5^ School of Health Sciences University of Southampton Southampton UK

**Keywords:** critical care, delirium, family‐centred care, family‐led interventions, interpretive descriptive

## Abstract

**Background:**

Interpretive descriptive design is a well‐established methodology in healthcare research, offering novel insights into complex phenomena that may elude other qualitative methods. Although interpretive descriptive is effective in translating findings into clinical practice, its application to intensive care unit interventions remains limited. The intricate nature of the intensive care unit environment and the vulnerability of this patient group, necessitates methodologies that delve deeply into interactions and translate them into practical solutions.

**Objective:**

This study applies an interpretive descriptive methodology to evaluate the Family Member's Voice Reorientation (FAMVR) programme, focusing on its impact on ICU patients, families and nurses and assessing its feasibility for clinical integration in delirium care.

**Methods:**

The study involved semi‐structured interviews with three patients, six family members and a focus group of eight nurses. The data were analysed thematically, revealing insights into how interpretive descriptive methodology can guide actionable insights into intensive care unit‐based, family‐led interventions.

**Findings:**

Interpretive descriptive methodology enabled a nuanced exploration of the FAMVR programme, capturing how it influenced the emotional, cognitive and practical aspects of care for patients, families and clinicians. The study found that sensitivity to context was crucial in understanding participants' experiences within the high‐stakes intensive care unit setting. The interpretive descriptive approach also allowed for a holistic view, incorporating physical, emotional, social and spiritual dimensions of care, thereby enhancing the intervention's relevance and applicability in delirium management.

**Conclusions:**

This study demonstrates that interpretive descriptive methodology is particularly well‐suited for evaluating complex intensive care unit interventions, providing a comprehensive understanding of participants' experiences and yielding practical, family‐centred recommendations. Findings suggest that interpretive descriptive methods can inform the refinement of interventions like FAMVR, making them more responsive to the unique needs of ICU patients and their families, while highlighting the value of family involvement in enhancing patient care. These insights position interpretive descriptive as a valuable approach for future qualitative research in healthcare settings.

**Patient or Public Contribution:**

No patient or public contribution.

**Trial Registration:**

ACTRN12622001568707; ANZCTR (https://www.anzctr.org.au/Trial/Registration/TrialReview.aspx?id=385122&isClinicalTrial=False)

## 1 Introduction

An interpretive descriptive qualitative study design is a methodological approach to conducting research that focuses on understanding people's experiences and how they make sense of them. Previously published studies have used interpretive descriptive qualitative design to understand the experiences of patients, nurses and health phenomena (Bulndi et al. 2023; Gurné et al. 2021; Timmins et al. 2023). In the context of intensive care units (ICU), where patients, families and clinicians navigate highly charged emotional and medical landscapes, interpretive descriptive methods offer a unique lens through which to evaluate interventions like the family member's voice reorientation (FAMVR) programme (Johnson, Towell‐Barnard, McLean, Robert, and Ewens 2024). This paper presents the FAMVR programme not as the primary subject of study but as an exemplar to illustrate the utility of interpretive descriptive methodology in clinical research.

By focusing on the methodological insights derived from this study, we aim to demonstrate how interpretive descriptive design can effectively capture the interactions and contextual meanings experienced by diverse participants in an ICU setting, offering practical, context‐specific recommendations for clinical practice. This methodological focus aligns with the broader goal of enhancing patient care and family collaboration through the lens of applied health research.

### 1.1 Qualitative Methodologies

Qualitative research approaches have become widely accepted as empirical research methods within health sciences, providing deeper and more detailed insights into human behaviour, experiences and phenomena (Jones et al. 2022; Pyo et al. 2023; Renjith et al. 2021). Qualitative approaches are particularly valuable because they prioritise depth over breadth, exploring complex issues involving human perceptions, emotions and interactions (Long and Jiang 2023; Pyo et al. 2023). Qualitative approaches focus on understanding the meaning and context of human experiences and can then use participatory methods to explore potential solutions (Busetto et al. 2020; Long and Jiang 2023). The evolution of qualitative research has given rise to a range of different methodological approaches within qualitative or mixed‐methods research paradigms (LaMarre and Chamberlain 2022; Renjith et al. 2021; Tomaszewski et al. 2020). These approaches include ethnography, grounded theory, phenomenology, narrative inquiry and interpretive descriptive (Renjith et al. 2021; Thorne 2011). Each of these methodologies has unique principles rooted in the qualitative philosophical paradigm (Renjith et al. 2021; Teherani et al. 2015). A paradigm is a framework of beliefs, values and methods that guides how researchers understand and investigate a particular field or phenomenon (Renjith et al. 2021; Teherani et al. 2015). Central to these philosophical paradigms are interpretivism, constructivism and critical theory, each providing a distinct approach to exploring and understanding human experiences and social phenomena (Creswell 2015; Paradis‐Gagné and Pariseau‐Legault 2022).

The philosophical stance in interpretivist approaches is that reality is socially constructed and understood best through personal experiences. This reaffirms the importance of context and the meanings associated with human actions and interactions (Ryan 2018). The interpretivist stance argues that reality is fluid and dynamic, not objective and is formed by those who experience it (Ryan 2018). Constructivism extends the interpretivist paradigm by focusing on how humans construct their realities through social interactions (Eads 2023; Peck and Mummery 2023). The constructivist stance argues that knowledge is generated between human participants and researchers, emphasising the dynamic and iterative process involved in this interaction (Peck and Mummery 2023). Within the constructivist paradigm, reflexivity is viewed as an essential process whereby researchers continuously reflect on their influence on the research process, whilst allowing the emergence of deep insights that are based on the lived experiences of the research participants (Khuzaiyah et al. 2023; Skukauskaite et al. 2022). Critical theory seeks to understand human experiences and takes this further by challenging and transforming societal constructs (Paradis‐Gagné and Pariseau‐Legault 2022). This range of research paradigms informs qualitative research methods, guides research design and the interpretation of the findings. Therefore, selecting a suitable approach is critical to research methodology.

### 1.2 Interpretive Descriptive (ID) Methodology

The Interpretive Descriptive (ID) method was developed by Thorne et al. (1997) and addresses the gap between purely descriptive qualitative research methodologies and theory‐focused approaches. In this study, the ID approach was chosen to explore the intricate experiences of patients, families and nurses interacting with the FAMVR programme. ID principles are grounded in both the interpretivist and constructivist paradigms, recognising that human reality is constructed through their experiences and interactions (Ocean et al. 2022; Thompson Burdine et al. 2021). ID focuses on the generation of new understandings that can inform clinical practice, making it a well‐suited approach to applied health research (Thorne et al. 1997, 2004). Compared to other qualitative methodologies that focus mainly on participants' narratives, ID emphasises the creation of coherent descriptions that are contextually grounded and have direct implications for clinical practice (Thorne 2011). This study was additionally underpinned by a Person‐Centred Care (PCC) framework, which emphasises respecting and responding to individuals' values, preferences and needs within the healthcare context (Santana et al. 2018). The PCC lens supported the interpretive process by ensuring that patient and family experiences were understood and applied in a way that enhanced personalisation, engagement and practical relevance of the intervention.

Despite the increasing emphasis on PCC in critical care settings, ICU patients often endure disorientation, anxiety and communication breakdowns due to the severity of their illness, sedation and restricted visitation (Kiwanuka et al. 2019). Family voices are seldom formally integrated into ICU care, despite their potential to support patient orientation and psychological well‐being (Vitorino et al. 2025). While PCC advocates for interventions that recognise individual needs, preferences and emotional safety, there remains a significant gap in how structured, family‐led voice interventions can operationalise PCC principles in delirium prevention and ICU care. This study seeks to address this gap by evaluating the FAMVR programme using an Interpretive Descriptive Approach, exploring both its implementation and its alignment with PCC principles.

One of the features of ID is purposive sampling, whereby participants are selected based on the richness and depth of their interactions and contextual insights relevant to the research question, and which ensures deep insights into the phenomenon being explored (Thorne et al. 2004). In‐depth interviews and focus groups are commonly used for data collection, allowing flexibility to explore participants' perspectives in detail and adjust data collection strategies as new insights emerge (Ocean et al. 2022). Interactive and interpretive analysis approaches are applied in ID, ensuring that data analysis remains focused on understanding participants' interactions and the contextual meanings of their perspectives (Thorne et al. 2004). Like other qualitative methods, the steps in data analysis in ID involve generating initial codes of the data and identifying similarities and themes which are then refined to form the final themes (Thompson Burdine et al. 2021). ID is distinct in its ability to generate descriptive and interpretive findings, offering a comprehensive account of the phenomenon under study while uncovering its deeper meanings (Ocean et al. 2022; Timmins et al. 2023). This methodological approach emphasises the practical implications of research findings, ensuring they are accessible and readily translatable into clinical practice for health care practitioners (Bulndi et al. 2023; Thorne et al. 2004). In this study, ID enabled the research team to move beyond superficial descriptions of participant experiences to uncover deeper themes related to emotional resilience, cognitive recovery and family involvement in care. These insights were not merely descriptive but were translated into actionable recommendations for refining the FAMVR intervention, demonstrating the practical utility of the ID methodology.

The analytical approach in ID is particularly notable for its commitment to producing contextually relevant, practically valuable and theoretically informed insights into participants' interactions and the meanings derived within their specific contexts (Thorne 2016; Timmins et al. 2023). This is especially valuable when studying complex health conditions, such as implementing a familiar voice orientation program to prevent delirium. Interpretive descriptive distinctiveness focuses on a practical understanding of participants' interactions and contextual meanings through an inductive and iterative process, which builds from the data rather than preconceived hypotheses. It is characterised by a sensitivity to the context, such as the ICU environment, and the generation of meaningful themes (Thorne 2016). The method also involves eliciting rich, detailed examples of participants' interactions and contextual meanings, and balancing theoretical insights with practical utility (Gurné et al. 2021; Thorne 2016). For example, a study exploring the essential characteristics of contemporary nursing practice from registered nurses' perspectives achieved substantial, contextually relevant findings. The nurses' descriptions of striving to work in close proximity to patients provided deeper insights into the responsibilities and roles of nurses, thereby clarifying current nursing practices and guiding the refinement and advancement of the profession (Gurné et al. 2021). Similarly, Bulndi et al. (2023) utilised ID to create a culturally safe space for women to explore and articulate their interactions and contextual meanings related to obstetric fistula risk factors and treatment. The interpretive analysis revealed insights that informed policy development and raised community awareness, enhancing maternal health outcomes in that region.

### 1.3 Case Study: The FAMVR Programme

The family member's voice reorientation (FAMVR) programme serves as a case study for the application of interpretive descriptive methodology. This programme, designed to provide auditory orientation via recorded messages from family members to critically ill patients, was co‐developed with ICU stakeholders, including patients, families and clinical staff (Johnson, Towell‐Barnard, McLean, Robert, and Ewens 2024). In this paper, we use the FAMVR programme as an example to illustrate how interpretive descriptive methods can be applied to evaluate the feasibility and acceptability of family‐led interventions in critical care settings.

The detailed clinical findings of the FAMVR intervention are reported elsewhere (Johnson, Towell‐Barnard, McLean, and Ewens 2024a). This paper highlights how the methodology facilitated a deep understanding of the participants' subjective perspectives and their interactions with the intervention. The FAMVR programme offers a practical illustration of how ID design can inform the iterative refinement of interventions, ensuring that they are both clinically relevant and sensitive to the emotional and social contexts of ICU patients and their families.

The FAMVR intervention involved family members recording short, structured voice messages on an iPad, co‐designed with former ICU patients, family members and clinical staff using the Double Diamond model to ensure collaborative development. The intervention comprised four domains: general orientation (played three times daily), ICU routines, procedures and a free category for personalised family updates. Messages provided reassurance, procedural context and emotional connection, each lasting 1–3 min. Family members were provided with a template and guidance to ensure consistency of the processes, and nurses facilitated playback of messages at appropriate times. A full description of the intervention and its development is available elsewhere (Johnson, Towell‐Barnard, McLean, and Ewens 2024a).

To ensure fidelity and feasibility of the intervention, four structured training sessions were delivered to the ICU nurses. These sessions provided a refresher on delirium, instruction on using the Richmond Agitation‐Sedation Scale (RASS), and step‐by‐step guidance on implementing the FAMVR protocol (Johnson, Towell‐Barnard, McLean, and Ewens 2024a). The RASS was used to assess patient readiness for voice playback, with a target range of 0 to −2, indicating optimal responsiveness. Nurses were also trained to support family members in recording messages and to identify appropriate times during care routines to deliver the recordings.

### 1.4 Methodology Selection: Key Considerations

The primary study was a mixed‐methods pilot study to determine the relationship between the intervention and patient outcome and the feasibility and acceptance of it. As a small‐scale pilot study, a qualitative approach can add depth and richness to the quantitative data (LaMarre and Chamberlain 2022; Renjith et al. 2021). The complexity of the ICU environment whilst integrating an intervention that involves a vulnerable population warrants detailed exploration, and ID is suitable for this context as participants' perspectives may vary significantly (Alshehri et al. 2022; Ruppel et al. 2019). Using the ID approach ensures that the meanings that participants attribute to the intervention are explored in depth and understood in the clinical context of the intervention to better enable its refinement and incorporation into practice (Alshehri et al. 2022; Julian et al. 2023; Turgut et al. 2022). ID also helps the researcher understand the participants' perspectives within their specific contexts, allowing for diverse experiences, which is vital in refining the intervention to address different demographic and patient populations (Turgut et al. 2022). ID moves from categorising data into themes as employed in other qualitative designs to focus on interpreting the contextual meanings and patterns of participants' perspectives, leading to deeper insights into the impact of the intervention (Ruppel et al. 2019). The clinical expertise of the researcher is essential to the ID approach because it enables the researcher to maintain reflexivity throughout the process, continually reflecting on their biases and perspectives, which contributes to the rigour and originality of the evaluation process (Houghton et al. 2012; Thorne et al. 2004).

### 1.5 Aims

To evaluate the family member's voice reorientation (FAMVR) programme using interpretive descriptive qualitative methods, focusing on its impact on intensive care unit patients, family members and nurses, and its feasibility and potential for clinical integration into delirium care. This study also aims to explore how interpretive descriptive methodology can be used to generate actionable insights into ICU interventions, thus contributing to the refinement of family‐led delirium management strategies.

## 2 Methods

### 2.1 Settings

The primary study was conducted in a 32‐bed general adult ICU in the United Kingdom (UK) (Table 1). The interpretive descriptive (ID) approach was well‐suited to this setting due to the complexity of working with critically ill patients experiencing delirium, which demanded a sensitive and flexible methodology capable of capturing rich, context‐specific insights. The ICU setting was selected to provide clinical relevance to the application of ID in evaluating the FAMVR programme, allowing findings to be directly translated into clinical practice and support timely improvements in patient care and experience (Dijkstra et al. 2023; Handberg and Voss 2018; Ruppel et al. 2019). The contextual challenges of the ICU environment, such as heightened emotional responses and cognitive impairments in patients, were integral to understanding how the FAMVR intervention could be practically implemented and adapted in such a setting. Consultation with key ICU stakeholders ensured that delirium management was identified as a priority and that a new intervention would be welcomed, fulfilling a core aspect of the ID approach (Thompson Burdine et al. 2021).

**TABLE 1:** Overview of the primary study and the role of ID in the evaluation stage.

Stages	Methods	Timeline	ID relationship
Problem exploration	Clinical experience Literature review (Johnson, Towell‐Barnard, McLean, and Ewens 2024b)	≥ 10 years December 2021–August 2023	The understanding gained from clinical experiences and the literature review was essential for framing the research question that would be addressed using ID later in the study.
Project administration	Research proposal Ethical approvals Study site setup	May 2021–March 2022 March 2022–March 2023 March 2023–April 2023	These steps ensured the study was ethically sound and properly structured, which was crucial for the later ID analysis.
Intervention development	A co‐design qualitative study (Johnson, Towell‐Barnard, McLean, Robert, and Ewens 2024)	April 2023–June 2023	The qualitative data gathered during co‐design helped shape the ID questions and guided the later ID analysis.
Intervention implementation	Single‐arm convergent mixed‐methods pilot (Johnson, Towell‐Barnard, McLean, and Ewens 2024a)	June 2023–February 2024	The qualitative data gathered here provided a foundation for the ID approach, which was used to interpret and understand the intervention's outcomes.
Intervention evaluation (this study)	Interpretive descriptive qualitative	February 2024–April 2024	This stage was where ID's strength in combining descriptive and interpretive findings was fully utilised, helping to generate actionable insights for clinical practice.

### 2.2 Participants

Participants were patients and family members aged 18 years and over who participated in the FAMVR programme in the pilot phase and voluntarily consented to participate in the evaluation stage after discharge from the hospital. The clinical group consisted of nursing staff working with patients receiving the FAMVR programme and providing care in the ICU. Given the focus on capturing rich, contextual insights, a purposive sampling strategy was employed to ensure the inclusion of participants with deep and diverse insights into the phenomenon under investigation. This was essential within the ID approach to guarantee that the resulting data would provide sufficient depth and breadth to refine the intervention (Dolan et al. 2023; Thompson Burdine et al. 2021).

To address potential selection bias, the researcher was cognisant to include a range of participants with varied demographic characteristics, ICU experiences and roles in the intervention (e.g., primary and secondary caregivers, junior and senior nurses). Patients and families had experienced different ICU trajectories, including short‐ and long‐term lengths of stay; nurses were selected from both day and night shifts to reflect different caregiving dynamics. Although participation was voluntary, recruitment was monitored to ensure variation in lived experience of ICU, which helped to mitigate homogeneity and enhance the diversity of the perspectives captured.

Evaluation occurred immediately after the FAMVR's implementation to ensure that participants could recall their experiences with clarity and accuracy.

### 2.3 Recruitment and Ethical Considerations

A purposive sampling strategy was employed to recruit participants, ensuring that individuals with knowledge of the phenomenon were recruited (Elliott and Timulak 2021; Thorne et al. 2004). This sampling method, integral to the ID approach, ensured that participants could contribute nuanced, context‐specific perspectives, enhancing the credibility and depth of the study findings (Thorne 2016).

#### 2.3.1 Recruitment of Patient and Family Participants

During the implementation phase of the primary study, eligible patients and family participants provided informed written consent that included their contact information. Participants were selected based on their involvement with the FAMVR programme to ensure that their feedback would be directly relevant to the evaluation of the intervention. The researcher monitored patient discharges via the electronic medical record (EMR) system and contacted participants within 1 month post‐discharge to arrange a mutually convenient time for the one‐to‐one interviews.

#### 2.3.2 Recruitment of Nurse Participants

With the support of a nurse educator, nursing staff were informed about the study and what participation would involve via the staff group page on WhatsApp and email. To ensure that the evaluation reflected the perspectives of those who had actively engaged with the FAMVR intervention, the nurse recruitment strategy focused on those with direct experience in its implementation. The researcher attended staff meetings and visited the ICU to provide further information as required. Data sufficiency was reached after the first focus group, indicating that the perspectives gathered were comprehensive enough to meet the study's aims.

#### 2.3.3 Ethical Approval

Ethical approval was obtained in March 2023 from the Health Research Authority (HRA) and Health and Care Research Wales (HCRW), and Research Ethics Committee, Project ID: 318820, REC reference: 23/LO/0057. Reciprocal ethical approval was obtained from the university where the author is a doctoral candidate. Special care was taken to mitigate any potential distress when participants recounted their ICU experiences, particularly given the vulnerability of this population. Interviews and focus groups were conducted in comfortable environments post‐hospital discharge, ensuring that participants had time to recover emotionally before discussing their experiences. All participants provided informed written consent.

### 2.4 Data Collection and Analysis

One‐to‐one audio‐recorded interviews were conducted with patient and family participants. Nursing staff attended a focus group, which was digitally recorded via Microsoft Teams and a dictaphone as backup. The decision to engage each group separately was made to capture their unique perspectives, ensuring that the experiences of patients, families, and nurses were explored without the influence of external viewpoints. This approach allowed the researcher to immerse themselves in the participants' contextual perspectives, maintaining the depth required by ID methodology (Dijkstra et al. 2023; Forozeiya et al. 2019). All interviews and focus group discussions were transcribed verbatim and coded using NVivo transcription software (Lumivero 2023; Naeem et al. 2023). Data collection focused on capturing detailed accounts of participants' perceptions, acknowledging the complexity and variability of individual experiences (Thompson Burdine et al. 2021).

The analysis process adhered to the principles of ID methodology, ensuring that the thematic analysis was grounded in the data while remaining open to interpretive insights that could inform practical improvements to the FAMVR intervention (Braun and Clarke 2006, 2020). All interviews and the focus group were transcribed verbatim and imported into NVivo for data management. The following steps were taken: (1) Transcription and initial reading: All transcripts were read multiple times to gain familiarity with the data and to begin noting early impressions. (2) Open coding: Initial line‐by‐line coding was conducted, focusing on identifying meaningful units of text that captured participants' experiences, perceptions and interactions with the intervention. Codes were generated inductively, reflecting participants' language and contextual nuances. (3) Collating and reviewing codes: Related codes were grouped together into broader conceptual clusters. A codebook was iteratively developed and refined during this process. (4) Theme development: Clusters of codes were reviewed and abstracted into higher‐order themes that represented recurring patterns across participants. Themes were reviewed against the original data to ensure coherence and relevance.

(5) Interpretation and contextualisation: Final themes were interpreted considering the ICU context, including clinical realities and emotional complexity. Reflexivity was maintained throughout by journalling analytic decisions and discussing theme development with supervisors. Themes were mapped against elements of the FAMVR intervention to identify implications for its refinement.

### 2.5 Rigour

Rigour is crucial to ID qualitative design (Thompson Burdine et al. 2021). Lincoln and Guba's (1985) concepts of trustworthiness, which include credibility, transferability, dependability, confirmability and authenticity, were applied to ensure the quality and trustworthiness of the study. Credibility was enhanced by drawing from a diverse participant group, ensuring that data reflected a range of perspectives on the FAMVR programme. The depth of analysis was further supported by dedicating ample time to interviews and focus groups, which allowed participants to express their perspectives fully.

Transferability was ensured by providing detailed descriptions of the study's context and processes, allowing findings to be applied to similar settings. A comprehensive audit trail was maintained, documenting decisions made throughout the research process to guarantee dependability. Reflexivity was employed to ensure that the researcher remained conscious of their biases, journaled reflections, and engaged in regular peer discussions to prevent undue influence on the findings (Johnson et al. 2020; Skukauskaite et al. 2022).

All researchers are qualified in ICU or social science, with varying clinical experience, and possess extensive knowledge and expertise in qualitative research. This background informed both their sensitivity to the ICU context and their interpretation of participant narratives. This clinical proximity, while beneficial for contextual understanding, also carried the risk of assumptions based on professional familiarity. To mitigate this, the researcher maintained a reflexive journal throughout the study, regularly interrogating their assumptions, documenting preconceptions and reflecting on how their role, identity, and values might shape interactions with participants and data interpretation. Peer debriefing with academic supervisors further helped to challenge and validate the researcher's interpretations, strengthening confirmability.

Member checking was adopted to ensure the accuracy of the data, with participants verifying that their perspectives had been accurately captured and interpreted. This process contributed to the confirmability and overall trustworthiness of the study (Johnson et al. 2020). During member checking, the researcher ensured that data belonging to other participants were not inadvertently revealed to others, thereby applying pseudonyms and maintaining the ethical principles of confidentiality.

## 3 Findings

### 3.1 Participant Description

A total of 17 participants (three patients, six family members and eight nurses) participated in the current study (Table 2). The diverse participant pool was purposively selected to provide comprehensive insights into the FAMVR intervention in alignment with the principles of interpretive descriptive methodology, which values depth and context over sample size.

**TABLE 2:** Patient demographics.

Participant group	Number of participants	Gender (male/female)	ICU experience (nurses only)
Patients	3	2 Males, 1 female	N/A
Family members	6	3 Males, 3 females	N/A
Nurses	8	3 Males, 5 females	4 with ≥ 5 years, 4 with ≥ 6 months

The sample size was adequate to achieve data sufficiency, as the participant narratives provided a diverse array of contextual and experiential insights necessary for evaluating and refining the intervention. Interpretive Descriptive research does not prioritise statistical generalisability; instead, it focuses on generating findings relevant to practice that can be applied to similar clinical settings. However, we acknowledge that a larger sample could yield additional perspectives, and future studies should consider expanding the participant pool to explore broader patterns and enhance the transferability of findings across various ICU environments.

### 3.2 Analysis of Themes From the Interpretive Descriptive Design

Seven key themes were identified from the thematic analysis, including acceptance of the FAMVR, cognitive state, communication, delirium awareness, perception of the ICU, reactions to the FAMVR, and well‐being outcomes. The interpretive descriptive approach not only facilitated the identification of these themes but also enabled an in‐depth understanding of how participants' perspectives varied within the ICU setting. By focusing on context and flexibility, ID allowed the research team to capture both similarities and differences in these perspectives. For example, the theme of communication and orientation was supported by family reflections describing how the patient responded to the familiarity of the voice, stating, ‘He's been listening to me for 50‐odd years’, which highlighted the calming and orienting effect of the recordings (Table 3). Table 3 illustrates the analysis of these themes, while Table 4 demonstrates their practical application for refining the intervention.

**TABLE 3:** Analysis of themes identified through the interpretive descriptive design.

Examples of participants' quotes	Transcription and initial reading	Coding	Developing themes	Interpretation and contextualisation
‘I think in the morning at some point we forgot to give handover and the paper is still there’. [Nurse]	Challenge in implementation	Handover challenges	Acceptance and utilisation challenges	The quote highlighted a practical issue in implementing the FAMVR intervention: the potential for forgetfulness and the inefficiency of paper‐based documentation.
‘They get overwhelmed because the environment is new to them. They are thinking, why? Why are they admitted in intensive care, how sick they are? And, I think they're just scared sometimes, especially when those alarms are going on and sometimes they're also confused on what's going on’. [Nurse]	Perception of patient confusion	ICU environment causing confusion	Cognitive state and environmental factors	The quote illustrates the disorienting effect of the ICU environment on patients, contributing to feelings of confusion and fear.
‘How come he did that for you? And I just said, isn't it very nice. The familiarity of the voice. I said, He's been listening to me for 50‐odd years’. [Family]	Family member's perspective	Family voice impact	Communication and orientation	The quote underscored how a family member's familiar voice can positively impact a patient's orientation and response.
‘I understood that X had two major kinds of delusions and I can't remember who told us that would be a result of medication’. [Family]	Family member's perspective	Delirium awareness from staff	Delirium awareness and communication	The quote revealed that the family was made aware of delirium symptoms and their possible connection to medication but indicates a gap in preparing them for ongoing care.
‘Also the way you do it, you don't try to have the impression that the teams are at the edge of something. It was very controlled and calm which I found really helpful’. [Family]	Family member's perspective	Calm and controlled environment	Perception of the ICU environment	The quote highlighted the family member's positive perception of the ICU environment, noting the staff's calm and controlled demeanour.
‘I've only noticed that they would open their eyes when they would hear the recording, which I really think is interesting they react to the voices of the relatives. So it's kind of cool feeling as well, It's good for your assessment as well. Like less effort to stimulate the patient’. [Nurse]	Nurse's observation	Patient reaction to family voices	Reactions to the FAMVR intervention	The quote highlighted that patients responded positively to the voices of their relatives, indicating an increased level of alertness.
‘I think at the moment the main goal as Y stated his goal, and I think we both want to get back to his goals and everything’. [Family]	Family member's perspective	Psychological recovery post‐discharge	Well‐being outcomes post‐discharge	The quote underscored the shared goal between the patient and the family member to return to pre‐illness activities and goals, indicating a focus on recovery and well‐being.

**TABLE 4:** Illustration of how interpretations helped to inform FAMVR clinical application.

Description	Interpretation	Application
‘I think in the morning at some point we forgot to give handover and the paper is still there’. [Nurse]	Insufficient resources affected the feasibility of the intervention.	Integration into electronic medical records to increase efficiency.
‘They get overwhelmed because the environment is new to them. They are thinking, why? Why are they admitted in intensive care, how sick they are? And, I think they're just scared sometimes, especially when those alarms are going on and sometimes they're also confused on what's going on’. [Nurse]	Environmental influences in the ICU contribute to the cognitive state of patients.	Strengthening and reinforcing family voice orientation.
‘How come he did that for you? And I just said, isn't it very nice. The familiarity of the voice. I said, He's been listening to me for 50‐odd years’.	Family member's voice orientation was beneficial to patient communication.	Consistent family member's voice integration in delirium care.
‘I understood that X had two major kinds of delusions and I can't remember who told us that would be a result of medication’.	Delirium information at the time of admission can promote awareness but not preparation for delirium symptoms.	Focusing delirium training and education on family members, including signs and symptoms of delirium, in the education.
‘Also the way you do it, you don't try to have the impression that the teams are at the edge of something. It was very controlled and calm which I found really helpful’.	Coordinated team approach enhanced family members' experience.	Integrating simplified and standardised approaches to delirium care, such as the FAMVR intervention.
‘I've only noticed that they would open their eyes when they would hear the recording, which I really think is interesting they react to the voices of the relatives. So it's kind of cool feeling as well, It's good for your assessment as well. Like less effort to stimulate the patient’. [Nurse]	Applying the FAMVR intervention before nursing assessments improves patient response and minimises painful stimulation.	Leveraging the FAMVR intervention in nursing assessment and patient care.
‘I think at the moment the main goal as Y stated his goal, and I think we both want to get back to his goals and everything’. [Family]	Participants experienced good psychological recovery; however, some challenges delayed their psychological recovery.	Implementing ICU recovery clinics to follow up with patients and family members post‐hospital discharge.

### 3.3 Analysis of Interpretive Descriptive Design to Evaluate the FAMVR

The use of the ID method was critical for evaluating the FAMVR intervention within the ICU context. This approach enabled the researcher to maintain a balance between analysing individual participant perspectives and synthesising shared themes across groups, ensuring both depth and breadth in understanding the intervention's impact. The methodology enabled a nuanced exploration of how the FAMVR affected patients, families and nurses, allowing for the simultaneous analysis of emotional, cognitive and practical aspects of the intervention. This multi‐dimensional approach is a key strength of interpretive descriptive methodology, providing a holistic understanding of complex healthcare interventions.

### 3.4 Sensitivity to Context

The ID method's emphasis on context allowed the study to capture how patients, family members and nurses interacted with the FAMVR intervention in the emotionally charged ICU environment. This contextual sensitivity is vital in healthcare research, as it ensures findings are not abstracted from the realities of clinical practice. As one family member noted:

Also the way you do it, you don't try to have the impression that the teams are at the edge of something. It was very controlled and calm which I found really helpful. [Family]

### 3.5 Focus on Contextual Perspectives

The ID methodology prioritises the subjective, contextual perspectives of participants, ensuring that their unique perspectives on the FAMVR programme were captured. This focus on individual narratives was essential in understanding how different participants interpreted and interacted with the intervention, allowing the researcher to tailor the programme to meet varying needs. For example, a family member reflected:

I do remember becoming emotional while I was doing it. But I'm glad, I did feel that it was worthwhile. [Family]

### 3.6 Flexibility

The inherent flexibility of the ID approach was key in responding to the unpredictable and rapidly evolving nature of ICU care. This flexibility enabled the researcher to adapt data collection strategies as new insights emerged, ensuring the study remained responsive to the participants' dynamic perspectives. One family member shared:

Obviously, it was very distressing, especially when she was first admitted and it was traumatic. [Family]

### 3.7 Holistic Understanding

The ID approach allowed for a comprehensive exploration of how the FAMVR programme contributed to delirium prevention and management, recognising that the participants' perspectives involved not only physical but also emotional, social and spiritual dimensions. This holistic understanding is critical in an ICU where care involves addressing multiple facets of human experience. By using ID, the study captured how these dimensions interacted within the ICU context, revealing deeper insights into the intervention's impact on patient security and emotional well‐being. One nurse highlighted the significance of the FAMVR in promoting a sense of safety for patients:

I have witnessed some of them who were responding to those personal voices from their family, so I can definitely see that has made them more secure. [Nurse]

This finding illustrates how ID methodology facilitates the integration of diverse experiential dimensions into intervention evaluation, making the results more practically applicable to care settings.

### 3.8 Respect for Individual Narratives

The ID methodology was particularly valuable in giving voice to patients and families who might otherwise feel powerless or unheard in a complex clinical environment. By prioritising individual narratives, the study captured personal stories that shaped the refinement of the FAMVR programme. This respect for unique perspectives ensured that the intervention was not only evaluated for its clinical effectiveness but also for its alignment with the emotional and psychological needs of the participants. As a family member reflected:

Nobody gave me any information about anything like that. So we just kept visiting every day. We were there as long as we could and praying. [Family]

This narrative reveals a communication gap that the FAMVR intervention helped address, showcasing how ID can uncover critical emotional and social factors that influence the success of ICU interventions. This finding highlights how ID methods can guide the refinement of interventions to support optimal family‐centred care.

### 3.9 Capacity to Capture Complexity

ICU contexts are characterised by a complex interplay of emotions, decisions and interactions between patients, families, and medical staff. The strength of ID methodology lies in its ability to capture this complexity, moving beyond surface‐level findings to uncover the nuanced experiences that shape intervention outcomes. The study revealed how FAMVR influenced communication dynamics in the ICU, as exemplified by one family member who shared:

There was a doctor on the Saturday and a doctor on the Sunday. And they said two completely different things. It was like two completely different viewpoints. And that was a bit disconcerting. [Family]

This example illustrates how the ID approach facilitated an understanding of communication inconsistencies and how the FAMVR programme could help mitigate these issues by providing a consistent and reassuring point of contact through the familiar voice. The complexity of ICU care requires interventions that can bridge communication gaps, and the ID methodology was instrumental in identifying these critical factors.

### 3.10 Attention to Cultural and Social Contexts

The ID methodology enabled the study to explore how cultural and social factors influenced the participants' perspectives on the FAMVR programme. These contexts played a significant role in shaping how patients and families coped with the ICU environment and the recovery process. By acknowledging these influences, the ID approach ensured that the intervention was evaluated not only for its technical or clinical aspects but also for its relevance to the participants' broader social and cultural realities. One participant shared how the programme fostered family support during a critical time:

It certainly affected my boys, but I liked how they felt. Well, we just thought that was it, basically, that was the end of the road. And they talk among themselves about it. [Family]

This finding highlights how the FAMVR programme not only aided in patient orientation but also strengthened family bonds and provided a shared experience that helped families navigate the emotional challenges of critical illness. The intervention's value was partly rooted in cultural expectations of close‐knit family roles and intergenerational caregiving, which shaped how families engaged with and interpreted their involvement. Some participants expressed a spiritual or emotional framing of the experience, suggesting that their coping was embedded in shared family narratives, beliefs and rituals. These social and cultural layers influenced the meaning attributed to the recordings and how families viewed their responsibility to contribute. Future refinements of the intervention should consider tailoring content and engagement strategies to reflect diverse cultural norms surrounding family involvement, voice and health communication. The attention to cultural and social contexts, facilitated by the ID methodology, made it possible to tailor the intervention to better support both patients and their families in coping with the ICU experience.

### 3.11 Refinement of the FAMVR Intervention

The findings from the ID analysis informed several key refinements of the FAMVR intervention. The iterative, inductive process of the ID method allowed the researcher to generate insights that directly influenced the intervention's practical application. For instance, embedding documentation within the electronic medical record system was identified as a strategy to improve the programme's integration into clinical practice. Through the lens of ID methodology, the findings emphasised the need for ongoing patient orientation to reduce anxiety and confusion. The intervention refinement focused on ensuring that family members' voices were consistently used to provide this orientation, thus supporting patients in responding more positively to treatment. The ID process also highlighted the importance of preparing family members to recognise the signs of delirium, emphasising the need for targeted education while recording the voice messages. This methodological insight shows how ID can bridge the gap between intervention design and clinical implementation.

### 3.12 Experiences of Participants

Participants expressed that their involvement in the study was a fulfilling experience. The ID approach's emphasis on flexibility and contextual understanding helped create an environment where participants felt safe to share personal and complex reflections on their experiences with the FAMVR intervention. This supportive framework allowed family members, patients and nursing staff to openly express not only appreciation but also critical insights that contributed to refining the intervention. For example, a family member remarked:

Well, can I just say it was a pleasure to meet and talk to you. [Family]

Another patient and family member also expressed their appreciation for the experience:

It is lovely to see you again, and thank you for all the help you gave us. While you were there, it was absolutely amazing. We really did appreciate it. And so did the boys. [Family]

A participant further expressed their positive experience of the nursing staff:

The nursing staff in the intensive care and HDU were in fact incredibly professional and caring, very compassion at the same time. [Family]

The participatory nature of ID also encouraged staff to reflect on their professional practices in a structured, focused way, which they found valuable for self‐assessment. One nurse expressed gratitude for this reflective opportunity:

Thank you for the time and it was lovely to have this group. We were just talking and speaking about it. [Nurse]

### 3.13 Benefits and Challenges of ID in the FAMVR Intervention

The ID methodology proved particularly valuable in this study by enabling an in‐depth evaluation of the FAMVR intervention as a programme within the ICU setting. As a flexible, context‐sensitive approach, ID was instrumental in identifying both practical and clinical gaps in delirium care that structured, hypothesis‐driven methodologies might have overlooked. One of the key strengths of the ID design is its flexibility and sensitivity to context, allowing the study to identify gaps in delirium care that more structured methodologies might have overlooked. For example, one nurse reflected on the lack of standardised delirium care tools:

I think we have a gap in delirium. And then it is also, how do we prevent that? So if we have a standardised tool for us to prevent it, I mean, even if not fully preventable. But at least we're doing something. [Nurse]

This reflection exemplifies interpretive descriptive flexible approach allowed the research team to identify areas for improvement in clinical practice that might be neglected by more rigid evaluation frameworks. The ID design was also beneficial in capturing the practical integration of the FAMVR intervention into nursing routines and understanding how it influenced nurses' behaviours and decision‐making processes.

One of the distinctive advantages of using ID for programme evaluation is its focus on generating actionable insights rather than testing predefined outcomes. By capturing rich, contextual insights, the ID approach allowed the development of a logic model to refine the intervention for future studies and potential incorporation into broader clinical practice. For instance, a nurse described how the FAMVR programme positively impacted patient care and assessment:

When we have a long‐term patient admitted into ICU when you play it, especially when you're doing the rolling or washing the patient, then I could see on their faces that they're more calm like they are more adherent. Like you could tell them to turn right. They are more keen on doing that. [Nurse]

This observation illustrates how the ID methodology provided practical insights into how the intervention affected patient behaviour, offering direct, context‐driven guidance on refining the programme to enhance its effectiveness. By contrast, traditional programme evaluation approaches, such as experimental or quasi‐experimental designs, often focus on specific outcome measures without allowing the same depth of contextual exploration.

However, applying the ID methodology in this study presented several challenges, particularly in ensuring methodological coherence and maintaining sufficient participation throughout the data collection process. Given that the FAMVR intervention was introduced to 30 participants during the pilot phase, it was crucial to ensure that the ID stage included a representative sample of those participants. The researcher had to carefully manage the follow‐up process to ensure key discharge dates were recorded; interviews were conducted at suitable times, maintaining an audit trail to track these interactions.

Another notable limitation of using ID in programme evaluation is the challenge of synthesising diverse perspectives within a complex healthcare context. Integrating the diverse perspectives of patients, family members and nurses posed a challenge, as their views on the delirium intervention varied significantly. A nurse participant, for example, emphasised that despite extensive training in delirium practices, there remained gaps in how this knowledge was applied in clinical settings:

Delirium is like a very broad topic and it's very important in ICU patients, we have been given lots of training because I'm doing an ICU course right now. So we have been taught as well. [Nurse]

In contrast, a family member's perspective highlighted a disconnect between medical training and communication with families:

All I knew is that Z was in ICU and that very few people came out of it. That was all I knew. Nobody gave me any information about anything like delirium. [Family]

Synthesising these diverse insights was challenging, as the interpretations varied across participant groups. However, this complexity also provided valuable insights into the need for improved delirium awareness training, particularly for family members. Aligning the training of healthcare professionals with better communication and education for families would help bridge this gap. While interpretive descriptive emphasis on contextual and individualised insights is highly beneficial, the approach can sometimes make it difficult to achieve a coherent, unified model for programme improvement, a task that more structured programme evaluation approaches might handle more easily.

Reflecting on the benefits and limitations of using ID for programme evaluation, this study highlights the importance of continuous reflexivity, flexibility and strategic planning. Although ID lacks the standardisation of other evaluation methods, it compensates by providing nuanced, context‐specific recommendations that are directly applicable to the intervention setting. For complex interventions like FAMVR, this adaptability is critical, as it allows the programme to be refined based on feedback that is both practical and responsive to the immediate needs of patients, families and clinicians.

## 4 Discussion

This paper demonstrates the value of interpretive descriptive (ID) methodology in evaluating complex clinical interventions, using the FAMVR programme as an exemplar. While the clinical outcomes of the programme are important, the primary focus here is on the methodological insights gained from using ID in an ICU setting. Interpretive descriptive methods captured the nuanced, context‐specific perspectives of ICU patients, families, and nurses, generating a rich dataset that informed the evaluation and refinement of the FAMVR programme. The flexibility of the ID approach was crucial in enabling the research team to adapt to the dynamic and often unpredictable nature of ICU care while maintaining methodological coherence and rigour.

One of the most significant benefits of ID methodology was its ability to identify patterns and themes that were not immediately apparent, such as the impact of family voices in promoting patient orientation and psychological recovery. These insights highlight the importance of methodological flexibility and reflexivity in applied health research, particularly when working with vulnerable populations in high‐stakes environments like the ICU. By capturing these intricate, contextual insights, ID ensured that the findings were directly translatable into actionable steps for improving patient care. Future research can build on these methodological insights by applying ID methods to other ICU‐based interventions, ensuring that the contextual perspectives of patients and families remain central to the development and evaluation of family‐centred care strategies. The emphasis on capturing real‐time, context‐specific data allows for immediate application to practice, supporting more responsive and adaptive healthcare interventions.

The importance of family‐centred care identified in this study is consistent with wider literature, where family participation is recognised as crucial to improving patient outcomes in ICU settings (Dijkstra et al. 2023). Additionally, family‐centred care has been shown to support the emotional and psychological coping mechanisms of both patients and relatives beyond the ICU environment (Lemetti et al. 2021). While family‐centred care is well established in paediatric care (McCarthy and Guerin 2022; Ridgway et al. 2021), its transferability to adult ICU settings may vary (Bohart et al. 2023). By applying ID to a family‐led intervention, this study has provided valuable insights that can be applied directly to ICU delirium care while offering a transferable framework for exploring similar phenomena in other healthcare contexts (Dolan et al. 2023; Handberg and Voss 2018; Thompson Burdine et al. 2021). This approach aligns with broader literature highlighting the importance of family participation in patient care (Hochendoner et al. 2022; Wong et al. 2020).

Although the ID approach is widely utilised as a qualitative design (Bulndi et al. 2023; Julian et al. 2023; Timmins et al. 2023), the challenges of applying ID in ICU interventions remain underreported. One of the most significant challenges is maintaining methodological coherence. If the ID framework is not applied consistently and logically, there is a risk that the findings may not translate effectively into clinical practice (Hunt 2009; Thorne et al. 2004; Thompson Burdine et al. 2021). In this study, the researcher's reflexivity and attention to potential biases were crucial to maintaining the integrity of the methodology. Adequate reflection on the researcher's influence on the data collection and interpretation process is vital, as any oversight could undermine the validity of the findings (Thorne et al. 2004; Thorne 2016).

While the literature has addressed some challenges associated with using the ID method (Hunt 2009; Wiesner 2022), this study contributes novel insights by applying ID within the unique context of ICU patients and a digital family‐led delirium intervention. This application provides an important case for how ID can be used in similar high‐pressure, complex healthcare environments where clinical, emotional and social factors interact dynamically.

The findings also demonstrate that the FAMVR intervention operationalises key PCC principles by integrating family involvement directly into the patient's care experience, providing emotional reassurance, familiar orientation cues, and support for clinical recovery. In doing so, this study contributes to closing a significant gap in PCC application within ICU delirium management, illustrating how personalised family input can enhance care delivery and patient‐centred outcomes in this vulnerable population.

### 4.1 Strength and Limitation

A significant strength of this study is its use of ID methodology to capture and interpret the contextual perspectives of patients, family members and nurses involved in a digital family‐led delirium intervention. The interpretive descriptive approach ensured that the findings were deeply contextualised within the ICU setting, allowing for practical applications in real‐time clinical care for delirium. The categorisation of participant descriptions into actionable insights facilitated the refinement of the FAMVR intervention, demonstrating how ID can be used to bridge the gap between qualitative insights and practical implementation.

However, several limitations must be considered when interpreting the findings. One limitation of the study is that it does not encompass a broad range of patient, family and nursing staff experiences. Expanding the participant group could have captured a wider variety of perspectives, potentially revealing additional themes and insights. As the sample consisted of 17 participants from a single ICU site, the findings may not reflect the diversity of ICU environments or patient demographics found in other healthcare settings. This limits the transferability of the findings and the generalisability to broader ICU contexts.

Although data sufficiency was reached after the first focus group, it is possible that additional perspectives were not captured. This is particularly relevant to the nurse participants, where further focus groups might have uncovered more nuanced variations in practice or interpretation of the intervention. Further research incorporating multiple focus groups is recommended to broaden the range of insights and strengthen these findings.

Additionally, the perspectives of other healthcare professionals, such as medical doctors, physiotherapists, ICU psychologists and health support workers, were not captured in this study. Including these professional perspectives could have provided a more comprehensive understanding of the FAMVR intervention's impact and highlighted additional areas for intervention refinement. Their absence may have resulted in an incomplete picture of the intervention's integration within the multidisciplinary ICU context. Future research should explore these additional viewpoints, as they may offer unique insights into the ID approach's applicability across different professional roles and patient care practices.

## 5 Conclusion

Utilising interpretive descriptive methodology in this study provided a robust framework for evaluating the FAMVR programme and generating actionable insights that can enhance ICU care. This paper has highlighted how interpretive descriptive methods can capture complex human experiences in clinical settings, offering practical recommendations for intervention refinement. This methodological approach provides significant advantages for health researchers seeking to balance descriptive richness with clinical applicability. By focusing on the contextual, emotional and social dimensions of ICU care, the interpretive descriptive design facilitates a deeper understanding of how interventions like the FAMVR programme can be tailored to meet the needs of patients and families. Future studies applying this methodology will continue to advance the field of qualitative health research, providing valuable insights that can inform both practice and policy in critical care settings.

## Author Contributions

**Gideon U. Johnson:** conceptualisation, methodology, software, validation, formal analysis, investigation, resources, data curation, writing – original draft, writing – review and editing, visualisation, project administration. **Amanda Towell‐Barnard:** conceptualisation, methodology, validation, formal analysis, resources, writing – review and editing, supervision. **Christopher McLean:** methodology, validation, formal analysis, resources, writing – review and editing, supervision. **Glenn Robert:** methodology, validation, formal analysis, resources, writing – review and editing, supervision. **Beverley Ewens:** conceptualisation, methodology, validation, formal analysis, resources, writing – review and editing, supervision.

## Conflicts of Interest

The authors declare no conflicts of interest.

## Data Availability

The data that support the findings of this study are available on request from the corresponding author. The data are not publicly available due to privacy or ethical restrictions.

## References

[nop270291-bib-0001] Alshehri, H. H. , A. Wolf , J. Öhlén , and S. Olausson . 2022. “Healthcare Professionals' Perspective on Palliative Care in Intensive Care Settings: An Interpretive Descriptive Study.” Global Qualitative Nursing Research 9: 1–12. 10.1177/23333936221138077. PMC9729985 36507302

[nop270291-bib-0002] Bohart, S. , C. Lamprecht , A. S. Andreasen , T. Waldau , A. M. Møller , and T. Thomsen . 2023. “Perspectives and Wishes for Patient and Family Centred Care as Expressed by Adult Intensive Care Survivors and Family‐Members: A Qualitative Interview Study.” Intensive & Critical Care Nursing 75: 103346. 10.1016/j.iccn.2022.103346. 36470701

[nop270291-bib-0004] Braun, V. , and V. Clarke . 2006. “Using Thematic Analysis in Psychology.” Qualitative Research in Psychology 3, no. 2: 77–101. 10.1191/1478088706qp063oa.

[nop270291-bib-0005] Braun, V. , and V. Clarke . 2020. “Can I Use TA? Should I Use TA? Should I Not Use TA? Comparing Reflexive Thematic Analysis and Other Pattern‐Based Qualitative Analytic Approaches.” Counselling and Psychotherapy Research 21, no. 1: 37–47. 10.1002/capr.12360.

[nop270291-bib-0006] Bulndi, L. B. , S. Bayes , E. Adama , and D. Ireson . 2023. “North‐Central Nigerian Women's Experiences of Obstetric Fistula Risk Factors and Their Perceived Treatment Services: An Interpretive Description.” Women and Birth 36, no. 5: 454–459. 10.1016/j.wombi.2023.02.007. 36868989

[nop270291-bib-0007] Busetto, L. , W. Wick , and C. Gumbinger . 2020. “How to Use and Assess Qualitative Research Methods.” Neurological Research and Practice 2: 14. 10.1186/s42466-020-00059-z. 33324920 PMC7650082

[nop270291-bib-0008] Creswell, J. W. 2015. Research Design: Qualitative, Quantitative, and Mixed Methods Approaches. 4th ed. SAGE Publications.

[nop270291-bib-0009] Dijkstra, B. M. , K. M. Felten‐Barentsz , M. J. M. van der Valk , J. G. van der Hoeven , L. Schoonhoven , and L. C. M. Vloet . 2023. “Exploring Patients' and Relatives' Needs and Perceptions Regarding Family Participation in Essential Care in the Intensive Care Unit: A Qualitative Study.” Intensive & Critical Care Nursing 79: 103525. 10.1016/j.iccn.2023.103525. 37598505

[nop270291-bib-0010] Dolan, S. , L. Nowell , and N. J. Moules . 2023. “Interpretive Description in Applied Mixed Methods Research: Exploring Issues of Fit, Purpose, Process, Context, and Design.” Nursing Inquiry 30, no. 3: e12542. 10.1111/nin.12542. 36464974

[nop270291-bib-0011] Eads, R. 2023. “Navigating Post‐Trauma Realities in Family Systems: Applying Social Constructivism and Systems Theory to Youth and Family Trauma.” Australian and New Zealand Journal of Family Therapy 44, no. 2: 214–224. https://doi‐org.ezproxy.ecu.edu.au/10.1002/anzf.1531.

[nop270291-bib-0012] Elliott, R. , and L. Timulak . 2021. Essentials of Descriptive‐Interpretive Qualitative Research: A Generic Approach. American Psychological Association. 10.1037/0000224-000.

[nop270291-bib-0013] Forozeiya, D. , B. Vanderspank‐Wright , F. F. Bourbonnais , D. Moreau , and D. K. Wright . 2019. “Coping With Moral Distress – The Experiences of Intensive Care Nurses: An Interpretive Descriptive Study.” Intensive & Critical Care Nursing 53: 23–29. 10.1016/j.iccn.2019.03.002. 30948283

[nop270291-bib-0014] Gurné, F. L. , E. Lidén , E. J. Ung , M. Kirkevold , J. Öhlén , and S. Jakobsson . 2021. “Striving to Be in Close Proximity to the Patient: An Interpretive Descriptive Study of Nursing Practice From the Perspectives of Clinically Experienced Registered Nurses.” Nursing Inquiry 28, no. 2: e12387. 10.1111/nin.12387. 33108693 PMC8244039

[nop270291-bib-0015] Handberg, C. , and A. K. Voss . 2018. “Implementing Augmentative and Alternative Communication in Critical Care Settings: Perspectives of Healthcare Professionals.” Journal of Clinical Nursing 27, no. 1–2: 102–114. 10.1111/jocn.13851. 28401613

[nop270291-bib-0016] Hochendoner, S. J. , T. H. Amass , J. R. Curtis , et al. 2022. “Voices From the Pandemic: A Qualitative Study of Family Experiences and Suggestions Regarding the Care of Critically Ill Patients.” Annals of the American Thoracic Society 19, no. 4: 614–624. 10.1513/AnnalsATS.202105-629OC. 34436977 PMC8996268

[nop270291-bib-0017] Houghton, C. , A. Hunter , and P. Meskell . 2012. “Linking Aims, Paradigm and Method in Nursing Research.” Nurse Researcher 20, no. 2: 34–39. 23316536 10.7748/nr2012.11.20.2.34.c9439

[nop270291-bib-0018] Hunt, M. R. 2009. “Strengths and Challenges in the Use of Interpretive Description: Reflections Arising From a Study of the Moral Experience of Health Professionals in Humanitarian Work.” Qualitative Health Research 19, no. 9: 1284–1292. 10.1177/1049732309344612. 19690208

[nop270291-bib-0019] Johnson, G. U. , A. Towell‐Barnard , C. McLean , and B. Ewens . 2024a. “The Implementation and Evaluation of a Family‐Led Novel Intervention for Delirium Prevention and Management in Adult Critically Ill Patients: A Mixed‐Methods Pilot Study.” Nursing in Critical Care 30: e13210. 10.1111/nicc.13210. 39617957 PMC12224221

[nop270291-bib-0020] Johnson, G. U. , A. Towell‐Barnard , C. McLean , and B. Ewens . 2024b. “Delirium Prevention and Management in an Adult Intensive Care Unit Through Evidence‐Based Nonpharmacological Interventions: A Scoping Review.” Collegian 31, no. 4: 232–251. 10.1016/j.colegn.2024.05.001.

[nop270291-bib-0021] Johnson, G. U. , A. Towell‐Barnard , C. McLean , G. Robert , and B. Ewens . 2024. “Co‐ Designing a Digital Family‐Led Intervention for Delirium Prevention and Management in Adult Critically Ill Patients: An Application of the Double Diamond Design Process.” International Journal of Nursing Studies 160: 104888. 10.1016/j.ijnurstu.2024.104888. 39303642

[nop270291-bib-0022] Johnson, J. L. , D. Adkins , and S. Chauvin . 2020. “A Review of the Quality Indicators of Rigor in Qualitative Research.” American Journal of Pharmaceutical Education 84, no. 1: 7120. 10.5688/ajpe7120. 32292186 PMC7055404

[nop270291-bib-0023] Jones, T. , L. Enria , S. Lees , M. Marchant , and O. Tulloch . 2022. “Changing Gear: Experiences of How Existing Qualitative Research Can Adapt to an Unfolding Health Emergency.” Frontiers in Sociology 7: 958861. 10.3389/fsoc.2022.958861. 36386857 PMC9650412

[nop270291-bib-0024] Julian, P. , J. Ploeg , S. Kaasalainen , and R. M. Markle . 2023. “Building Collaborative Relationships With Family Caregivers of Hospitalized Older Persons With Delirium Superimposed on Dementia: A Qualitative Study.” Journal of Advanced Nursing 79, no. 8: 2860–2870. 10.1111/jan.15449. 36196458

[nop270291-bib-0025] Khuzaiyah, S. , K. H. Abdul‐Mumin , L. McKenna , and S. Haji Hashim . 2023. “The Importance of Reflexivity in Data Collection Methods for Qualitative Midwifery Research.” British Journal of Midwifery 31, no. 12: 701–705. 10.12968/bjom.2023.31.12.701.

[nop270291-bib-0026] Kiwanuka, F. , S. J. Shayan , and A. A. Tolulope . 2019. “Barriers to Patient and Family‐Centred Care in Adult Intensive Care Units: A Systematic Review.” Nursing Open 6, no. 3: 676–684. 10.1002/nop2.253. 31367389 PMC6650666

[nop270291-bib-0027] LaMarre, A. , and K. Chamberlain . 2022. “Innovating Qualitative Research Methods: Proposals and Possibilities.” Methods in Psychology 6: 100083. 10.1016/j.metip.2021.100083.

[nop270291-bib-0028] Lemetti, T. , E. Partanen , M. Hupli , and E. Haavisto . 2021. “Cancer Patients' Experiences of Realisation of Relatives' Participation in Hospital Care: A Qualitative Interview Study.” Scandinavian Journal of Caring Sciences 35, no. 3: 979–987. 10.1111/scs.12918. 33107636

[nop270291-bib-0029] Lincoln, S. Y. , and E. G. Guba . 1985. Naturalistic Inquiry. Sage.

[nop270291-bib-0030] Long, Q. , and H. Jiang . 2023. “Qualitative Research in Health: Value and Visibility.” Lancet Regional Health. Western Pacific 34: 100790. 10.1016/j.lanwpc.2023.100790. 37283968 PMC10240377

[nop270291-bib-0031] Lumivero . 2023. “NVivo (Version 14).” www.lumivero.com.

[nop270291-bib-0032] McCarthy, E. , and S. Guerin . 2022. “Family‐Centred Care in Early Intervention: A Systematic Review of the Processes and Outcomes of Family‐Centred Care and Impacting Factors.” Child: Care, Health and Development 48, no. 1: 1–32. 10.1111/cch.12901. 34324725

[nop270291-bib-0033] Naeem, M. , W. Ozuem , K. Howell , and S. Ranfagni . 2023. “A Step‐By‐Step Process of Thematic Analysis to Develop a Conceptual Model in Qualitative Research.” International Journal of Qualitative Methods 22: 1–28. 10.1177/16094069231205789.

[nop270291-bib-0034] Ocean, M. , R. Montgomery , Z. Jamison , K. Hicks , and S. Thorne . 2022. “Exploring the Expansive Properties of Interpretive Description: An Invitation to Anti‐Oppressive Researchers.” International Journal of Qualitative Methods 21: 1–14. 10.1177/16094069221103665.

[nop270291-bib-0035] Paradis‐Gagné, E. , and P. Pariseau‐Legault . 2022. “Critical Research and Qualitative Methodologies: Theoretical Foundations and Contribution to Nursing Research.” Research and Theory for Nursing Practice 36, no. 2: 119–138. 10.1891/RTNP-2021-0014. 35584891

[nop270291-bib-0036] Peck, B. , and J. Mummery . 2023. “Hermeneutic Constructivism: One Ontology for Authentic Understanding.” Nursing Inquiry 30, no. 2: 1–10. 10.1111/nin.12526. 36283973

[nop270291-bib-0037] Pyo, J. , W. Lee , E. Y. Choi , S. G. Jang , and M. Ock . 2023. “Qualitative Research in Healthcare: Necessity and Characteristics.” Journal of Preventive Medicine and Public Health = Yebang Uihakhoe Chi 56, no. 1: 12–20. 10.3961/jpmph.22.451. 36746418 PMC9925284

[nop270291-bib-0038] Renjith, V. , R. Yesodharan , J. A. Noronha , E. Ladd , and A. George . 2021. “Qualitative Methods in Health Care Research.” International Journal of Preventive Medicine 12: 20. 10.4103/ijpvm.IJPVM_321_19. 34084317 PMC8106287

[nop270291-bib-0039] Ridgway, L. , N. Hackworth , J. M. Nicholson , and L. McKenna . 2021. “Working With Families: A Systematic Scoping Review of Family‐Centred Care in Universal, Community‐Based Maternal, Child, and Family Health Services.” Journal of Child Health Care 25, no. 2: 268–289. 10.1177/1367493520930172. 32602735

[nop270291-bib-0040] Ruppel, H. , M. Funk , R. Whittemore , S. Wung , C. P. Bonafide , and H. Powell Kennedy . 2019. “Critical Care Nurses' Clinical Reasoning About Physiologic Monitor Alarm Customisation: An Interpretive Descriptive Study.” Journal of Clinical Nursing 28, no. 15/16: 3033–3041. 10.1111/jocn.14866. 30938915

[nop270291-bib-0041] Ryan, G. 2018. “Introduction to Positivism, Interpretivism and Critical Theory.” Nurse Researcher 25, no. 4: 14–20. 10.7748/nr.2018.e1466. 29546962

[nop270291-bib-0042] Santana, M. J. , K. Manalili , R. J. Jolley , S. Zelinsky , H. Quan , and M. Lu . 2018. “How to Practice Person‐Centred Care: A Conceptual Framework.” Health Expectations 21, no. 2: 429–440. 10.1111/hex.12640. 29151269 PMC5867327

[nop270291-bib-0043] Skukauskaite, A. , I. Yilmazli Trout , and K. A. Robinson . 2022. “Deepening Reflexivity Through Art in Learning Qualitative Research.” Qualitative Research 22, no. 3: 403–420. 10.1177/1468794120985676.

[nop270291-bib-0045] Teherani, A. , T. Martimianakis , T. Stenfors‐Hayes , A. Wadhwa , and L. Varpio . 2015. “Choosing a Qualitative Research Approach.” Journal of Graduate Medical Education 7, no. 4: 669–670. 10.4300/JGME-D-15-00414.1. 26692985 PMC4675428

[nop270291-bib-0046] Thompson Burdine, J. , S. Thorne , and G. Sandhu . 2021. “Interpretive Description: A Flexible Qualitative Methodology for Medical Education Research.” Medical Education 55, no. 3: 336–343. 10.1111/medu.14380. 32967042

[nop270291-bib-0047] Thorne, S. 2011. “Toward Methodological Emancipation in Applied Health Research.” Qualitative Health Research 21, no. 4: 443–453. 10.1177/1049732310392595. 21164035

[nop270291-bib-0048] Thorne, S. 2016. Interpretive Description: Qualitative Research for Applied Practice. 2nd ed. Routledge. 10.4324/9781315545196.

[nop270291-bib-0049] Thorne, S. , S. R. Kirkham , and J. MacDonald‐Emes . 1997. “Interpretive Description: A Noncategorical Qualitative Alternative for Developing Nursing Knowledge.” Research in Nursing & Health 20, no. 2: 169–177. 10.1002/(sici)1098-240x(199704)20:2<169::aid-nur9>3.0.co;2-i. 9100747

[nop270291-bib-0050] Thorne, S. , S. R. Kirkham , and K. O'Flynn‐Magee . 2004. “The Analytic Challenge in Interpretive Description.” International Journal of Qualitative Methods 3, no. 1: 1–11. 10.1177/160940690400300101.

[nop270291-bib-0051] Timmins, J. L. , N. M. Kayes , and D. W. O'Brien . 2023. “Valuing Professional and Cultural Diversity in Support for Hand Therapists in Aotearoa New Zealand: An Interpretive Description Study.” New Zealand Journal of Physiotherapy 51, no. 1: 24–32. 10.15619/NZJP/51.1.04.

[nop270291-bib-0052] Tomaszewski, L. E. , J. Zarestky , and E. Gonzalez . 2020. “Planning Qualitative Research: Design and Decision Making for New Researchers.” International Journal of Qualitative Methods 19: 1–7. 10.1177/1609406920967174.

[nop270291-bib-0053] Turgut, Y. , H. Güdül Öz , M. Akgün , İ. Boz , and H. Yangın . 2022. “Qualitative Exploration of Nurses' Experiences of the COVID‐19 Pandemic Using the Reconceptualized Uncertainty in Illness Theory: An Interpretive Descriptive Study.” Journal of Advanced Nursing 78, no. 7: 2111–2122. 10.1111/jan.15153. 35099084

[nop270291-bib-0054] Vitorino, M. L. , A. Henriques , G. Melo , and H. R. Henriques . 2025. “The Effectiveness of Family Participation Interventions for the Prevention of Delirium in Intensive Care Units: A Systematic Review.” Intensive & Critical Care Nursing 89: 103976. 10.1016/j.iccn.2025.103976. 40024138

[nop270291-bib-0055] Wiesner, C. 2022. “Doing Qualitative and Interpretative Research: Reflecting Principles and Principled Challenges.” Political Research Exchange 4, no. 1: 2127372. 10.1080/2474736X.2022.2127372.

[nop270291-bib-0056] Wong, P. , B. Redley , R. Digby , A. Correya , and T. Bucknall . 2020. “Families' Perspectives of Participation in Patient Care in an Adult Intensive Care Unit: A Qualitative Study.” Australian Critical Care 33, no. 4: 317–325. 10.1016/j.acc.2019.06.002. 31371242

